# Development of a high throughput human stool specimen processing method for a molecular *Helicobacter pylori* clarithromycin resistance assay

**DOI:** 10.1371/journal.pone.0224356

**Published:** 2019-12-16

**Authors:** Natalie Clines, Erin Beckman

**Affiliations:** Meridian Bioscience, Inc., Cincinnati, Ohio, United States of America; Nitte University, INDIA

## Abstract

It has become critical to detect Helicobacter *pylori* (*H*. *pylori*) infection due to the link to gastric cancer with some strains. These strains are also increasing in resistance to antibiotics with clarithromycin leading the way as the first line treatment. Resistance to clarithromycin has been shown to correlate with the A2142G, A2142C, and A2143G mutations on the *rrl* gene. In the last few decades, non-invasive specimens, such as stool, have been a reliable alternate to gastric biopsy for immunoassay tests. More recently, it has been proven feasible for stool to be used in molecular based tests. Many of the core laboratories in the United States need a high throughput sample preparation to run this test. Here, a high throughput assay is compared to a previously published manual sample prep *H*. *pylori* molecular based assay. Using the Magna Pure 96 (Roche), at least 96 stool species and 96 biopsy specimens can be tested in an 8-hour shift of a clinical lab. The high throughput sample prep had a positive percent agreement (PPA) of 87% compared to the manual sample prep using the same testing configuration. The genotype predictions from the high throughput assay matched genotype predictions from the manual sample prep with the same stool sample 92% of the time. A concordance rate of 89% was observed with genotype predictions from the high throughput assay of the same patient stool and biopsy. In stool samples from the high throughput assay, there was 100% concordance between the quantitative polymerase chain reaction (qPCR)-derived genomic prediction and DNA sequencing data. The high throughput workflow can get more patients tested faster in addition to detection of mutations associated with clarithromycin resistance.

## Introduction

*Helicobacter pylori* has infected one-half of the world’s population [1]. It has become increasingly important to test for *H*. *pylori* infection as there is strong evidence showing a high correlation between infection and gastric cancer [2,3]. Although gold standard testing includes an invasive endoscopic procedure where a gastric biopsy is retrieved, there are tests where other specimen types can be used [4]. One commonly performed test is the urea breath test (UBT) in which the patient ingests a capsule containing ^13^C-labeled urea. Once the capsule is digested, the patient breathes into a capture vessel, releasing isotopic CO_2_ and ammonia [5]. The amount of CO_2_ released is measured against the patient’s baseline. The change in CO_2_ is used to determine if *H*. *pylori* is present in the stomach. There are also rapid immunoassay tests and ELISA assays available for *H*. *pylori* that involve collection and use of stool specimens [6,7]. Once the patient is diagnosed with an *H*. *pylori* infection, a regimen of antibiotics with a proton pump inhibitor (PPI) is prescribed. Typically, the first line of treatment includes the antibiotic clarithromycin (CLA) [8,9], although drug resistance to this antibiotic is increasing in prevalence [10]. The prevalence of clarithromycin resistance in the pediatric population in Italy, for example, was shown to be 25% from 2002 to 2007 [10]. It is important to detect resistance to minimize the number of repeat treatments and circumvent treatment failures. Currently, there are no FDA cleared tests for detecting *H*. *pylori* and CLA resistance using non-invasively collected specimens. A recently published manuscript described a novel molecular assay using analyte specific reagents (Meridian Bioscience, Cincinnati, Ohio) to amplify and detect *H*. *pylori* DNA in stool specimens as well as detect mutations associated with CLA drug resistance [11]. A manual sample preparation method was employed in that instance. There is a need for core laboratories to process a larger number of non-invasive *H*. *pylori* specimens in an automated fashion. The objective of this study was to use a *H*. *pylori* positive population to compare an optimized high throughput, semi-automated workflow to a reference manual workflow described previously with stool and stomach biopsy specimens.

## Materials and methods

### Samples and processing methods

Unpreserved stool and biopsy specimens were collected from patients who exhibited symptoms of *H*. *pylori* infection. Specimens were collected as part of the patients’ standard of care (symptomatic) in a private practice in Bologna, Italy and were de-identified upon testing. Specimens were stored at -20°C prior to testing. Mostly positive stool specimens were processed with the test method which was a high throughput, semi-automated sample preparation using the Roche MagNA Pure 96 (MP96). The MagNA Pure 96 DNA and Viral NA Small Volume Kit, Version 07, “Pretreatment of Stool Samples” was used and the instructions followed (cat no. 06543588001; Roche) for both the stool and biopsy specimens. The volumes used in this protocol were scaled down to correspond with 200 μL of sample transferred to the MagNA Pure 96 Processing Cartridge for on-board processing. The lysis buffer used for stool pre-treatment was MagNA Pure Bacteria Lysis Buffer (cat no. 04659180001; Roche). The pre-treatment protocol used for biopsy specimens is specified in the instructions for the MagNA Pure Tissue Lysis Buffer (cat no. 04805160001; Roche). This protocol recommends use of lysis beads, however, historical data indicates loss of this specific target during the mechanical homogenization and was, therefore, removed from the process. The homogenizer used was the Qiagen Tissuelyser II. For both specimen types, stool and tissue biopsy, 20 μl of the exogenous control (cat no. BIO-11025; Bioline) was added. The program run on the instrument, Pathogen Universal 200 3.1, was also the same for both specimen types. The sample input volume onto the MP96 was 200 μl with a corresponding 50 μl elution volume. One 96-well plate was tested for each specimen type containing a few select negative samples to detect any possible cross-contamination events.

### Reference testing

The reference manual method used was the QIAamp Fast Stool kit (Qiagen) following the manufacturer’s instructions for performing human DNA isolation. For this process, a qPCR exogenous control (cat no. BIO-11025; Bioline) was spiked into each stool sample prior to extraction which acted as a workflow performance control as well as in indicator of inhibition. Both test and reference testing were carried out at the same time. Biopsies, collected from symptomatic patients from the MP96 assay were compared to bidirectional sequencing and stool results from the same patient. Duplicate biopsy specimens were not available for reference testing with the manual method.

### Detection and analysis

All testing for *H*. *pylori* and CLA resistance was carried out using TaqMan real-time PCR amplification as described previously using the Qiagen RotorGene Q instrument [5]. A qPCR master mix was created using the following components: 2X SensiPLUS master mix (catalog no. BIO-11021; Bioline); *H*. *pylori* primer set (catalog no. ASR100; Meridian Bioscience) at a final concentration of 400 nM; *H*. *pylori* probe (cat no. ASR101; Meridian Bioscience) at a final concentration of 100 nM; *H*. *pylori* clarithromycin resistance (HPCR) probe (cat no. ASR102; Meridian Bioscience) at a final concentration of 100 nM; and control primers and probe (cat no. BIO-11025; Bioline) at a final concentrations of 40 nM and 100 nM, respectively. The *H*. *pylori* TaqMan probes’ binding sites were within the same amplified target on the *H*. *pylori rrl* gene. The Hp detection probe was detected in the green channel (emission wavelength, 510 nm) and the HPCR probe was detected in the red channel (emission wavelength, 660 nm). For the eluted samples processed using the MP96 workflow, 10 mg/mL BSA stock was added to a final concentration of 0.2 mg/ml in the qPCR master mix to reduce possible invalids due to interfering substances from stool. The volume of the MP96 processed samples added to the final qPCR mix was 10 μl. For specimens processed using the reference manual method, the addition of BSA was unnecessary and the sample volume input was 20 μl. Molecular-grade water was used in all reactions to adjust the final reaction volume to 50 μl. The following qPCR thermoprofile was used: 95°C for 5 min followed by 50 cycles each of 95°C for 20 seconds and 65°C for 60 seconds. The final step of the qPCR program was a melt step of the red probe (emission wavelength, 660 nm). The melt step was conducted between 50°C and 70°C with a 10 second wait between 1°C step increments.

**Fig 1:**
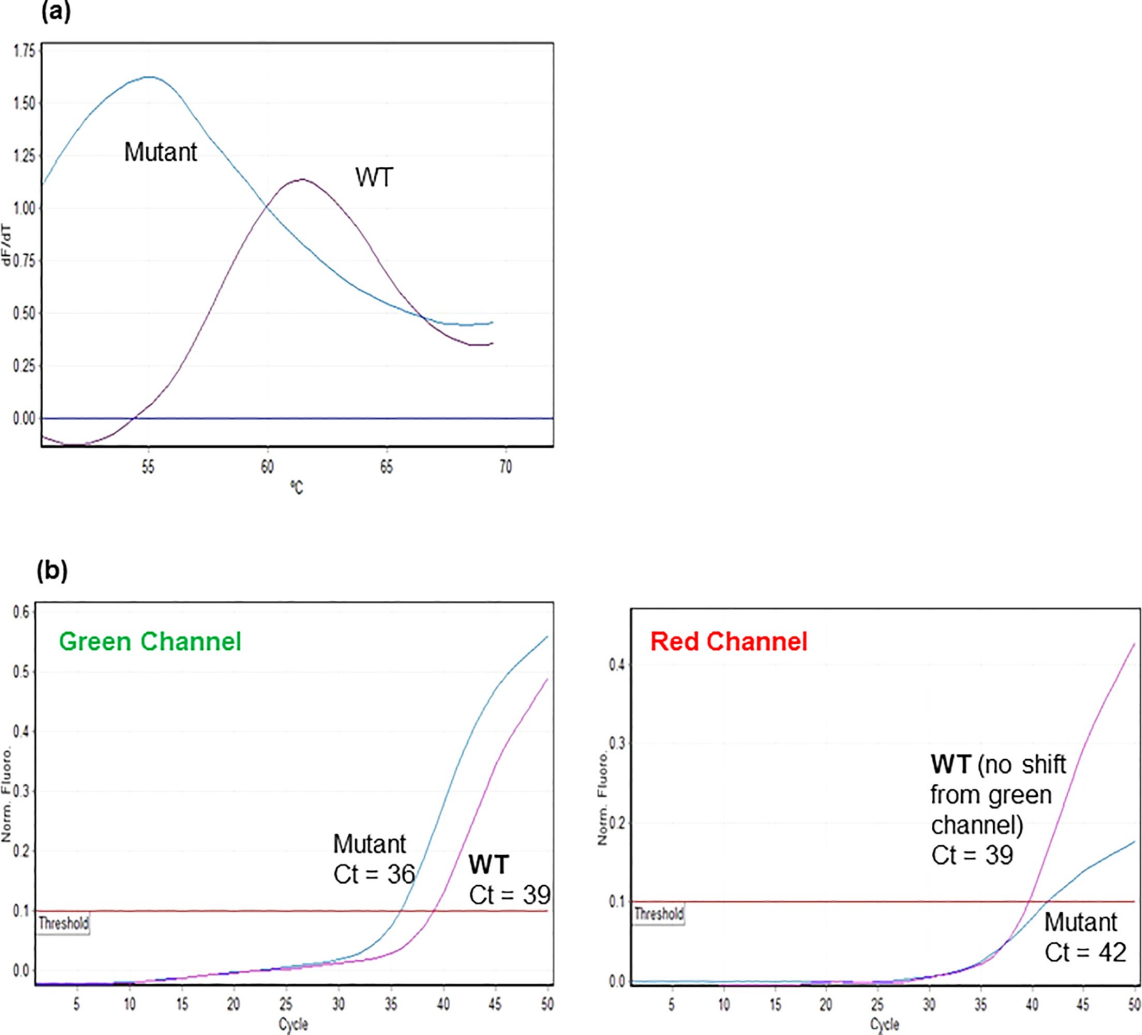
Melt curve analysis and observed changes in Ct values. (a) Analyzed melt curves for 2 specimens; 1 wild-type (purple curve) and 1 mutant (blue curve); (b) shift in Ct values for 2 samples, wild type (purple curve) and mutant (blue curve), red channel Ct minus green channel Ct.

The simultaneous detection of *H*. *pylori* and the prediction of CLA resistance/susceptibility was analyzed as previously described [11]. If amplification was observed in the green channel with the *H*. *pylori* probe, the specimen was positive. If no amplification crossed the set threshold of 0.1 normalized fluorescent units after 50 cycles, the sample was considered negative. If a specimen was positive for *H*. *pylori*, typically a single melt peak with the HPCR probe was observed. The RotorGene Q software would assign a single melt temperature (T_m_) using a heavy digital filter on the red (HPCR probe) signal. The presence or absence of a mutation associated with CLA resistance would show a different melt profile and T_m_ value so resistance or susceptibility can be predicted. The stool specimens for both the test and reference assays had a T_m_ cutoff of 60°C to indicate wild type (≥60°C; susceptible) or mutant (<60°C; resistant) (Fig 1). In the case of an indeterminate melt result, the change in Ct, also known as delta Ct, was analyzed to predict CLA resistance or susceptibility. The Ct value from the normalized red channel (HPCR probe) was subtracted from the Ct value of the normalized green channel (Hp probe). If the difference between Ct values was higher than a shift of 2 Ct values, the sample was considered CLA susceptible (Fig 1). Those samples with a Ct shift of less than 2 were considered CLA resistant.

For biopsy specimens from the MP96 high throughput assay, a similar algorithm was used to predict CLA resistance. The T_m_ cutoff for biopsy samples was different than that of stool. Biopsies were given a cutoff of ≥57°C for CLA sensitivity and <57°C for CLA resistance. Bidirectional sequencing was used to verify the absence or presence of the mutations associated with CLA resistance of *H*. *pylori* positive samples. Purification of the amplified DNA followed by bidirectional sequencing was performed by ACGT, Inc. (IL, USA). Sequences were aligned using Sequencher software (Gene Codes Corporation).

## Results

### Stool specimens

A total of 96 stool specimens were processed using the MP96 high throughput and reference manual methods. Table 1 shows the overall step-wise workflow of the high throughput method. Of the 96 specimens tested, 76 were positive and 20 were called negative by the manual reference assay. The MP96 assay had a positive percent agreement (PPA) of 86.8% with 66 positive confirmations compared to 76 by the manual reference method. The positive predictive value (PPV) of the MP96 assay was 92.7%.

**Table 1:** Duration time for each step of the high throughput, 96-well plate workflow.

**Hands off**	Possible Break Time		
**Hands on**			
**Semi-Automated High Throughput (Batch Size = 96)**	**Steps**	**Task**	**Approx. Duration**
	1	Input peanut size (100mg ± 10%) of raw stool in PBS/entire biopsy in TLB	30 min
	2	Homogenize (*excluded in manufacturer's procedure for BLB or TLB*)	1 min
	3	Centrifuge	5 s
	4	Add supernatant and BLB to Proteinase K^*a*^	10 min
	5	Heat treatment 70C^*a*^	10 min
	6	Heat treatment 95C^*a*^	10 min
	7	Transfer 200μl to MP process plate for **Roche MP96 Small Volume** kit	10 min
	8	Run **Pathogen Universal 100 3.1** (50μl)	1.5 h
	9	qPCR set up with BSA (10μl eluate in 50μl reaction)	1 h
	10	Run on 3 RotorGenes (36 reactions each) simultaneously^*b*^	2.5 h
	11	Hp analysis of 96 total reactions	1.5 h

^a^ For stool sample ONLY; no proteinase K or heat steps necessary for biopsy samples; therefore, final time for biopsy processing may be 30–35 min. shorter

^b^ Only 1 instrument is needed if using a 96 well plate capable platform

**Fig 2:**
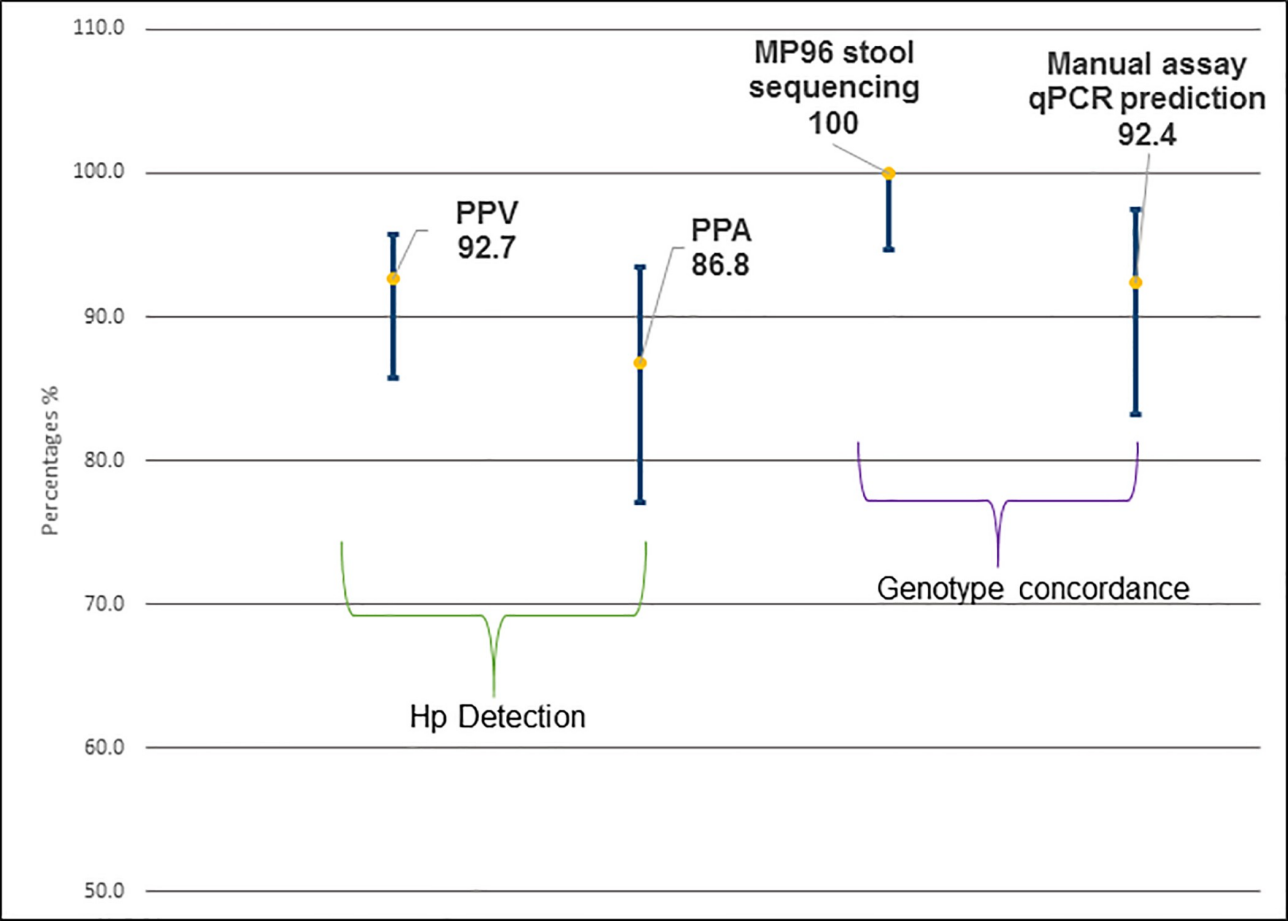
PPA and PPV results of MP96 stool assay compared to the reference manual stool assay of the same stool specimens for Hp detection; genotype concordance of the MP96 stool assay to sequencing and manual assay qPCR prediction.

*H*. *pylori* CLA resistance/susceptibility was also predicted and compared to the reference manual assay. After initial testing with the MP96, genotype predictions of 59 of the 66 positive specimens were concordant with manual reference testing. Of the 7 genotype discordant specimens, 5 were retested and were favorably resolved. Therefore, of the 66 positive specimens by the high through-put test assay, 61 were concordant on genotype prediction with the manual reference assay. Bidirectional sequencing was carried out on the 66 MP96 assay qPCR products. We observed a 100% concordance between the qPCR-derived genomic prediction and DNA sequencing data. The PPA and PPV rates had 95% confidence intervals compared to the reference assay for Hp (Fig 2). This plot includes concordance rates with a 95% confidence interval of the observed proportion for 66 *H*. *pylori* positive specimens from the MP96 assay compared to the manual reference assay qPCR predictions and the bidirectional sequencing results.

### Biopsy specimens

A total of 96 biopsy specimens from *H*. *pylori* symptomatic patients were tested with the MP96 test assay. Of the 96 specimens, 87 were positive for *H*. *pylori* and were bi-directionally sequenced. The sequencing results showed a 94% concordance rate compared to the biopsy MP96 qPCR assay. Some sequencing results showed either a mixed population result or inadequate sequencing results in 4 of the samples while 1 out of 87 samples did not match the qPCR genotype prediction for CLA resistance/susceptibility.

A subset of 32 positive biopsies had patient matched stools for further analysis. 4 results were indeterminate compared to the MP96 stool assay and 1 result was indeterminate compared to the manually processed stool assay. Of the 28 positive results, the genotype predictions of the biopsies versus that of the stool specimens from the MP96 stool assay and the reference manual stool assay were 89.3% (25/28) and 93.6% (29/31), respectively. There was a total of 5 total discordant specimens from the two comparator assays. Four out of the 5 discordant results were confirmed of their assay genotype prediction by their respective sequence result. One out of 5 discordant result was shown to be a mixed population after sequencing. Table 2 shows a breakdown of genotype concordance of the analyzed results.

**Table 2:** Matching patient genotype predictions from MP96 biopsy assay compared to MP96 stool assay and the reference manual stool assay.

Comparator/Reference Assay	No. of analyzed results	No. of Patients with concordant results (Stool v. Biopsy)	No. of Patients with discordant results (Stool v. Biopsy)	Rate of Concordance
MP96 Stool Assay	28	25	3	**89.3%**
Manual Stool Assay	31	29	2	**93.6%**

## Discussion/Conclusion

The results from stool and biopsy specimens show that sample prep automation is a feasible workflow for *H*. *pylori* detection and genotyping. Cutoff temperatures were different with stool compared to biopsy. This may be because less target is present in a stool sample due to degradation of free DNA. The difference in the amount of target resulted in a shift of the melt curves. Cutoffs were confirmed with bi-directional sequencing. The lower sensitivity shown by the high throughput assay could be due to the mechanism of DNA isolation from each method. The MP96 uses a silica bead to bind DNA under the optimal conditions, while the manual QIAamp method employs a silica membrane.

**Fig 3:**
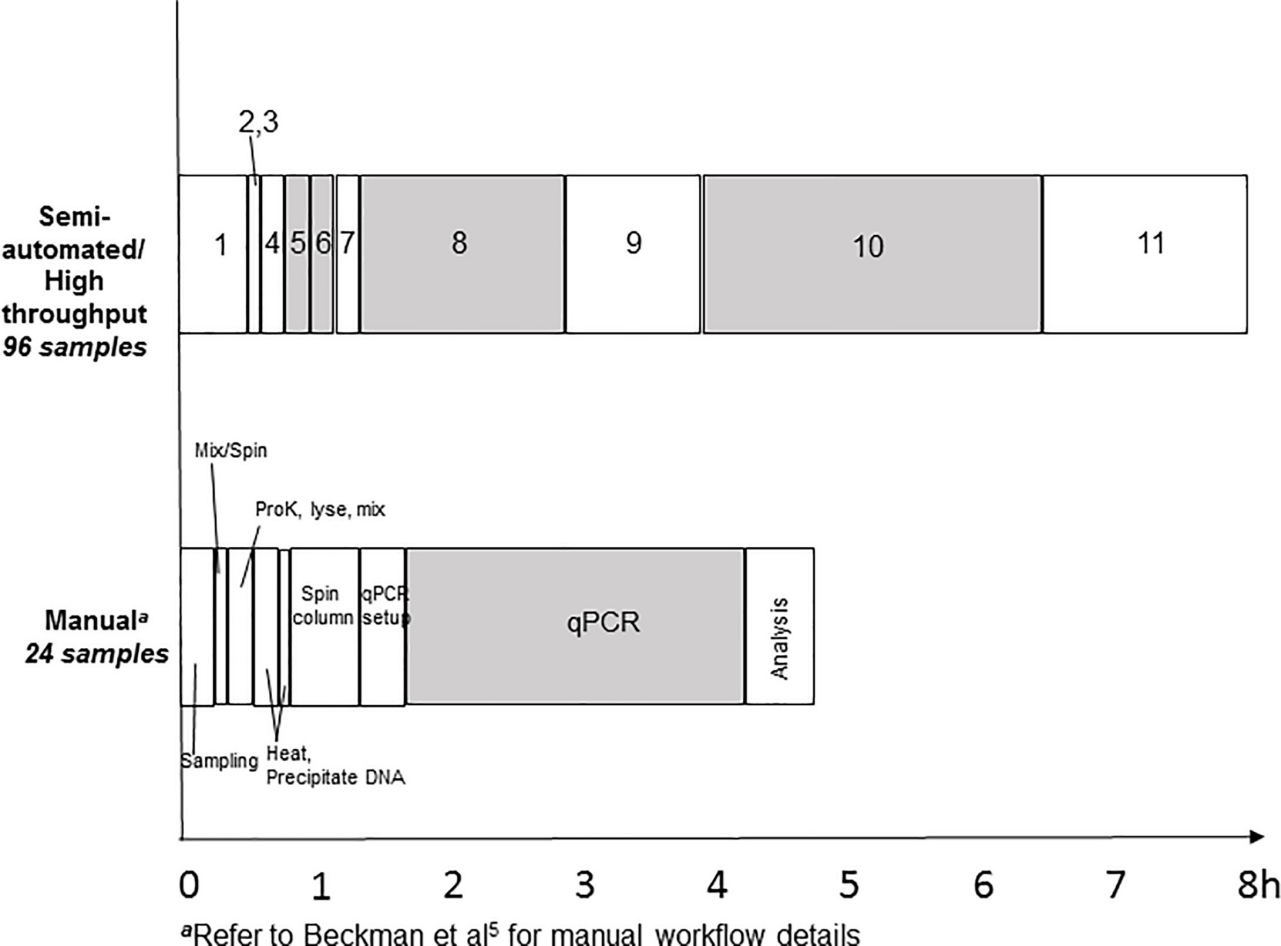
An 8-hour shift workflow for processing and analyzing 96 specimens from the Magna Pure 96 assay and 24 specimens from the manual assay. High throughput steps align with Table 1.

This study demonstrates that the number of specimens tested in a typical 8-hour shift can be scaled up; this includes any potential hands-off time for other laboratory tasks. An example of an 8-hour shift where the elapsed time for each task in the workflows are compared (Fig 3). For this study, the MP96 samples were processed off-board in individual tubes and then added to a 96-well processing plate to load on-board the MP96. If the appropriate equipment is available, an off-board processing in a deep well 96-well plate and a multi-channel pipette may reduce the risk of cross-contamination. Typically, a batch of 24 specimens is an ideal number for the manual method because it corresponds to the number of samples that most table top centrifuges can simultaneously handle. The stool specimens from the MP96 assay had a PPA of 87%. The PPV of stool specimens from the MP96 assay was 93%. The PPV was a biased result since the number of positive and negative stools is not an accurate indication of prevalence. With more negative specimens, the indication is that the PPV would increase.

It is well documented that three mutations on the *rrl* gene, A2142G, A2142C, and A2143G, are associated with CLA resistance [12,13]. Both stool and biopsy specimens showed a high concordance with respect to the sequencing results. *H*. *pylori* positive specimens can be infected with a mixed strain population [14,15]. Here, we observed a few cases that were confirmed by sequencing where one direction exhibited a wild type sequence and the other direction was a mutant sequence. These were categorized as indeterminate.

The high throughput, semi-automated assay using the MagNA Pure 96, is a reliable sample prep method for *H*. *pylori* testing in stool and biopsy specimens. It also can be used for the simultaneous prediction of CLA resistance.

## Supporting information

S1 Table Raw biopsy data. (XLSX)

S2 Table Raw stool data. (XLSX)
